# Frequent Gain and Loss of Introns in Fungal Cytochrome b Genes

**DOI:** 10.1371/journal.pone.0049096

**Published:** 2012-11-07

**Authors:** Liang-Fen Yin, Meng-Jun Hu, Fei Wang, Hanhui Kuang, Yu Zhang, Guido Schnabel, Guo-Qing Li, Chao-Xi Luo

**Affiliations:** 1 College of Plant Science and Technology and the Key Lab of Crop Disease Monitoring & Safety Control in Hubei Province, Huazhong Agricultural University, Wuhan, People’s Republic of China; 2 College of Horticulture and Forestry, Huazhong Agricultural University, Wuhan, People’s Republic of China; 3 School of Agricultural, Forestry & Environmental Sciences, Clemson University, Clemson, South Carolina, United States of America; University of Oxford, United Kingdom

## Abstract

In this study, all available cytochrome b (*Cyt b*) genes from the GOBASE database were compiled and the evolutionary dynamics of the *Cyt b* gene introns was assessed. *Cyt b* gene introns were frequently present in the fungal kingdom and some lower plants, but generally absent or rare in Chromista, Protozoa, and Animalia. Fungal *Cyt b* introns were found at 35 positions in *Cyt b* genes and the number of introns varied at individual positions from a single representative to 32 different introns at position 131, showing a wide and patchy distribution. Many homologous introns were present at the same position in distantly related species but absent in closely related species, suggesting that introns of the *Cyt b* genes were frequently lost. On the other hand, highly similar intron sequences were observed in some distantly related species rather than in closely related species, suggesting that these introns were gained independently, likely through lateral transfers. The intron loss-and-gain events could be mediated by transpositions that might have occurred between nuclear and mitochondria. Southern hybridization analysis confirmed that some introns contained repetitive sequences and might be transposable elements. An intron gain in *Botryotinia fuckeliana* prevented the development of QoI fungicide resistance, suggesting that intron loss-and-gain events were not necessarily beneficial to their host organisms.

## Introduction

Introns are widely distributed in numerous genes of viruses, prokaryotes and eukaryotes [1]–[4]. They are frequently found in mitochondrial genes of plant and fungal kingdoms, but are scarcely found in mitochondrial genes of Animalia, Protozoa and Chromista, although Animalia and Fungi are grouped together as Opisthokonts, and Chromista is phylogenetically close to Plantae [5]. Mitochondrial introns are classified into two main groups, group I and group II, based on their distinct RNA structures that facilitate their self-splicing activity [6]–[7]. Mitochondrial introns are usually group I introns and contain internal open reading frames (ORFs) which facilitate intron removal from RNA transcripts and intron propagation to new sites in genes [8]–[10].

Intron density in eukaryote genomes varies by more than three orders of magnitude, suggesting that there must have been extensive intron gain and/or loss events during evolution [11]. The evolutionary history of introns remains unresolved in many aspects. Several models have been proposed to explain the evolution and spread of introns. *Introns Late* theory argues that introns could have expanded in a fashion similar to transposable elements (TEs), have been the results of tandem duplications into exons [12], and were generated through a reverse-splicing mechanism catalyzed by the splicing machinery itself [13]. The presence of protein-encoding introns strongly supports the *Introns Late* theory where introns might be able to spread horizontally between phylogenetically distant species of different kingdoms [14]–[15].

In contrast, *Introns Early* theory suggests that introns were abundant in ancestral genes and mainly evolved through intron loss [16]–[17]. The prevailing theory for intron loss is that a processed mRNA is reverse transcribed to cDNA, which then recombines with the genomic copy of the gene, thereby precisely deleting the unmatched intronic sequence. This mechanism has been demonstrated experimentally in yeast [18] and many other studies also supported this mechanistic model [19]–[23]. Although the original concept of *Introns Early* vs *Late* was concerned with the origin of nuclear spliceosomal introns, the details of evolution of mitochondrial group I or group II introns could provide insights.

Most group I introns harbor genes encoding so-called homing endonucleases, initiating intron mobility *via* a double-strand breaks (DSBs)-repair process [24]. Many group I introns in organellar genomes encode maturases that help promote intron splicing by a variety of mechanisms [8], [25]. Some maturases also function *in trans* to promote splicing of other group I introns in the same genome [26]–[27]. The group II introns move *via* an RNA-based mechanism known as ‘retrohoming’. The proteins encoded by group II introns are multifunctional, containing maturase, reverse transcriptase and DNA-binding functions as well as DNA endonuclease activity. The maturase activity facilitates intron splicing by stabilizing the catalytically active RNA conformation, while other functions aid in the retrohoming to allelic target sites [28]–[29], as well as non-allelic sites [24], [30].

The number of mitochondrial introns or intron sequences may vary dramatically in different species. In recent studies on peach brown rot fungi *Monilinia* spp. (anamorphs in *Monilia*), *Cyt b* genes, the targets of the Quinone outside inhibitor fungicides (QoIs), were isolated and analyzed [31]–[33]. The *Cyt b* genes in *M. fructicola*, *M. laxa*, *M. fructigena* and *M. yunnanensis* have seven, whereas the *Cyt b* gene in *M. mumecola* has six large (>1 kb) introns. The intron/exon organization varies considerably among these species. In contrast to the large introns, the exons of *Cyt b* are usually small, ranging from 11 to 397 bp in length. The combined intron sequences of the *Cyt b* gene in *M. fructicola*, *M. fructigena*, *M. laxa*, *M. yunnanensis* and *M. mumecola* are 10,751, 12,380, 12,329, 11,456 and 13,027 bp, respectively. The large intron number and size make the *Cyt b* genes in *Monilinia* spp. the longest reported to date in all kingdoms of organisms. In contrast, the *Cyt b* gene in *Blumeria graminis* f.sp. *tritici*, the causal agent of wheat powdery mildew, is intronless, even though *Monilinia* and *Blumeria* species belong to the same fungal class Leotiomycetes. It will be interesting to know what caused the *Cyt b* genes in *Monilinia* species to harbor the largest ones in eukaryotic organisms.

The *Cyt b* genes have been sequenced and annotated from thousands of species including distantly related species from different kingdoms and widely used in systematic studies or evolutionary studies [34]–[40]. Furthermore, intron number and position in the *Cyt b* genes vary dramatically among different species. However, the dynamics of *Cyt b* gene introns, has not been systemically studied. In this study, we compiled all available *Cyt b* genes from the GOBASE database and assessed the evolutionary dynamics of *Cyt b* gene introns, and some introns in *Monilinia* and *Botryotinia* spp. were confirmed by Southern blot analyses.

**Table 1:** Distribution of introns in *Cyt b* genes of eukaryotic organisms.

Taxondivision^1^	No. of*Cyt b* genes^2^		Frequency of species with introns in the *Cyt b* gene (%)																	
	All	withintron	Unknown^3^		1 intron		2 introns		3 introns		4 introns		5 introns		6 introns		7 introns		Total	
			Spp.	%	Spp.	%	Spp.	%	Spp.	%	Spp.	%	Spp.	%	Spp.	%	Spp.	%	Spp.	%
Fungi	465	69	11	2.37	19	4.09	14	3.01	10	2.15	3	0.86	4	0.65	4	0.86	4	0.86	69	14.84
Metazoa	2062	3	0		3	0.15	0		0		0		0		0		0		3	0.15
Plants	440	9	0		2	0.45	3	0.68	4	0.91	0		0		0		0		9	2.05
Protista	309	11	1	0.32	6	1.94	2	0.65	1	0.32	1		0	0.32	0		0		11	3.56
Total	3276	92	12	0.37	30	0.92	19	0.58	15	0.46	4	0.12	4	0.12	4	0.12	4	0.12	92	2.81

1 In the GOBASE database, taxon divisions Fungi, Metazoa were equal to kingdoms Fungi, Animalia, respectively. Plant division represented the Plantae kingdom except for a few lower plants (i.e. some green algae). Protista included kingdoms Chromista and Protozoa, and some lower plants (green algae) which should have been in the kingdom Plantae based on the Cavalier-Smith’s classification system [5].

2 Data were obtained by gene search and intron search options with *Cyt b* gene (cob) as gene name in GOBASE database [41], and some of our own sequences were added, i.e. seven *Cyt b* introns from *M. fructicola*, *M. fructigena*, *M. laxa* and *M. yunnanensis*, six *Cyt b* introns from *M. mumecola* and *U. virens*.

3 The precise intron numbers were not known because these *Cyt b* genes were partial sequences. Spp. indicates biological species.

## Materials and Methods

### Isolation of *Cyt b* Gene from Rice False Smut Fungus *Ustilaginoidea virens*

The *Cyt b* genes were previously isolated from peach brown rot fungi *M. fructicola*, *M. fructigena*, *M. laxa*, *M. yunnanensis* and *M. mumecola* [31]–[33]. In the present study, the *Cyt b* gene was isolated from the rice false smut fungus *Ustilaginoidea virens*. Briefly, degenerate primers were designed based on *Cyt b* gene sequences from phylogenetically close species, and PCR reaction was carried out to amplify the conserved region from *U. virens* isolate UV-8a. Once a conserved fragment was isolated, the flanking sequences were obtained using Tail-PCR or Inverse-PCR amplifications and sequenced. For cDNA amplification, total RNA was isolated using the RNeasy Plant Mini Kit (Qiagen, Valencia, CA) according to the manufacturer’s instructions. First-strand cDNA was synthesized using an oligo-dT primer and Superscript III reverse transcriptase (Invitrogen, Carlsbad, CA) according to the manufacturer’s recommendations. Amplification of *Cyt b*-specific cDNA was performed using the primers designed to amplify the *Cyt b* sequence from translational start codon to translational stop codon. Intron numbers and precise locations were identified by comparing genomic DNA (gDNA) and cDNA sequences.

### Data Mining of *Cyt b* Gene Sequences

*Cyt b* gene and intron sequences were obtained directly from the GOBASE database (http://gobase.bcm.umontreal.ca/) using the gene search and intron search options with *Cyt b* gene (cob) as gene name [41]. Unfortunately, the GOBASE database has been stopped updating since June 2010 and the accession to the database (http://gobase.bcm.umontreal.ca/) is invalid currently. However, the accession nos. in GenBank (http://www.ncbi.nlm.nih.gov/) of all *Cyt b* genes showed in this study were indicated either in the table or figure legends.

**Table 2:** Characterization of locations of fungal *Cyt b* gene introns (positions and phases)^1^.

Intron locations corresponding to the *Cyt b* amino acid sequence of *Ustilaginoidea virens*																			
n.d^2^	52	67	69	93	95	97	114	117	122	126	131		137	138	139	141	143	146	160
En	Mp1(0)	Aa1(0)	Ad(2)	Gz1(2)	St1(0)	Mfg3(1)	Hj1(0)	Rs1(0)	Mv1(0)	Aj1(0)	Bf2(0)	Mn2(0)	Am2(0)	Sp2(0)	Sc1(1)	Sp3(1)	Am3(0)	Ptr2(0)	Mfn3(0)
		Am1(0)		Mfg2(2)		Mmj1(1)				Ms2(0)	Cb(0)	Mv2(0)	Hc(0)		Sd2(1)		Bc(0)		
		Bf1(0)				Mn1(1)				Pt2(0)	Cm(0)	My2(0)					Bf3(0)		
		Mfc1(0)								Rs2(0)	Cnd1(0)	Nc1(0)					Co(0)		
		Mfg1(0)								Sp1(0)	Cp1(0)	Pa2(0)					Cp2(0)		
		Ml1(0)									Db(0)	Pm(0)					Gi2(0)		
		Mm1(0)									Ef(0)	Pt3(0)					Mfc3(0)		
		Mp2(0)									Gi1(0)	Ptr1(0)					Mfn2(0)		
		Ms1(0)									Gz2(0)	Ro1(0)					Ml3(0)		
		My1(0)									Mfc2(0)	Rs3(0)					Mm3(0)		
		Pa1(0)									Mfg4(0)	Scs1(0)					My3(0)		
		Pb1(0)									Mfn1(0)	Sd1(0)					Sc2(0)		
		Pt1(0)									Mg1(0)	Spr1(0)					Scs2(0)		
		Ss(0)									Ml2(0)	Tp(0)					Sd3(0)		
		Uv1(0)									Mm2(0)	Um(0)					Yl1(0)		
		Wm(0)									Mmj2(0)	Uv2(0)							
1	1	16	1	2	1	3	1	1	1	5	32		2	1	2	1	15	1	1

Intron positions corresponding to the *Cyt b* amino acid sequence of *Ustilaginoidea virens*																		
163	164		168	169		171	188	200	206	208	229	253	260	263	270	275	289	386
Mv3(0)	Am4(1)	Mmj3(1)	Ac(0)	Ab(2)	Nf2(2)	Gi4(2)	Uv5(1)	Am5(0)	Scs3(1)	Hj2(2)	Pb2(0)	Am6(0)	Gz5(2)	Aj2(0)	Cbs2(2)	Aa2(1)	Mv5(2)	Mg3(0)
	Bf4(1)	Mn3(1)	Pn(0)	Gp2(2)	Sc3(2)	Rs4(2)		Mv4(0)			Spb(0)	Cnd3(0)	Mfc6(2)		Cc(2)	Mg2(1)	Rs6(2)	
	Cbs1(1)	My4(1)		Gz4(2)	Sd4(2)							Mfn5(0)	Mfg7(2)		Mfc7(2)	Ml7(1)	St3(2)	
	Cn(1)	Nc2(1)		Mfc5(2)	Spr2(2)							Sc4(0)	Ml6(2)		Sc5(2)	My7(1)		
	Cnd2(1)	Ro2(1)		Mfg6(2)	Tao2(2)							Yl3(0)	Mm6(2)		Yl4(2)	Pa3(1)		
	Gi3(1)	Ta(1)		Mfn4(2)	Tik(2)								My6(2)			Pc(1)		
	Gp1(1)	Tao1(1)		Ml5(2)	Uv4(2)											Rs5(1)		
	Gz3(1)	Td(1)		Mm5(2)	Yl2(2)											St2(0)		
	Mfc4(1)	Ti(1)		My5(2)												Tm(1)		
	Mfg5(1)	Tj(1)														Uv6(1)		
	Ml4(1)	To(1)																
	Mm4(1)	Uv3(1)																
1	24		2	17		2	1	2	1	1	2	5	6	1	5	10	3	1

1 Intron names consist of the first letter of the genus and the species and the intron number; intron phases are shown in parenthesis following intron names. For example, Mp1(0) indicated the first intron in *Moniliophthora perniciosa* and that it was a phase 0 intron, Ad(2) indicated the only phase 2 intron in *Ajellomyces dermatitidis*. In case the intron name was duplicated based on the above nomenclature, an additional letter was added for distinction, e.g. Mfc and Mfg introns, which indicated the introns from *Monilinia fructicola* and *Monilinia fructigena*, respectively. Phase 0 indicating an intron found between 2 codons, phase 1, between the first and second base of a codon, and phase 2, between the second and third base of a codon.

In brief, Aa: *Agrocybe aegerita* (AY781064), Ab: *Aspergillus brevipes* (AB026120), Ac: *Agrocybe chaxingu* (AY772389), Ad: *Ajellomyces dermatitidis* (ACBU01001794), Aj: *Aspergillus japonicas* (AF123598), Am: *Allomyces macrogynus* (NC_001715), Bb: *Beauveria bassiana* (EU371503), Bc: *Brettanomyces custersianus* (NC_013145), Bf: *Botryotinia fuckeliana* (AB428335), Cb: *Cordyceps brongniartii* (EU100743), Cbs: *Cryptococcus bacillisporus* (AB105919), Cc: *Candida castellii* (NC_012620), *Candida metapsilosis* (AY962591), Cn: *Cryptococcus neoformans* (AB105928), Cnd: *Candida neerlandica* (EU334437), Co: *Candida orthopsilosis* (AY962590), Cp: *Candida parapsilosis* (X74411), Db: *Dekkera bruxellensis* (NC_013147), Ef: *Epidermophyton floccosum* (AY916130), En: *Emericella nidulans* (J01388), Gi: *Glomus intraradices* (NC_012056), Gp: *Guehomyces pullulans* (AB175779), Gz: *Gibberella zeae* (DQ364632), Hc: *Hyaloraphidium curvatum* (NC_003048), Hj: *Hypocrea jecorina* (AF447590), Mfc: *Monilinia fructicola* (GQ304941), Mfg: *Monilinia fructigena* (HM149254), Mfn: *Millerozyma farinosa* (NC_013255), Mg: *Mycena galopoda* (X87997), Ml: *Monilinia laxa* (GU952817), Mm: *Monilia mumecola* (JN204425), Mmj: *Microdochium majus* (FJ560378), Mn: *Microdochium nivale* (FJ560375), Mp: *Moniliophthora perniciosa* (NC_005927), Ms: *Monoblepharella* sp. JEL15 (NC_004624), Mv: *Mycena viridimarginata* (X87998), My: *Monilia yunnanensis* (HQ908793), Nc: *Neurospora crassa* (M37324), Nf: *Neosartorya fischeri* (AY832916), Pa: *Podospora anserina* (X55026), Pb: *Paracoccidioides brasiliensis* (AY955840), Pc: *Podospora curvicolla* (AJ249985), Pm: *Penicillium marneffei* (AY347307), Pn: *Phaeosphaeria nodorum* (EU053989), Pt: *Pyrenophora teres* (DQ919067), Ptr: *Pyrenophora tritici-repentis* (DQ919068), Ro: *Rhizopus oryzae* (AY863212), Rs: *Rhizophydium* sp. (AF404306), Sc: *Saccharomyces cerevisiae* (AJ011856), Scs: *Smittium culisetae* (NC_006837), Sd: *Saccharomyces douglasii* (X59280), Sp: *Spizellomyces punctatus* (AF404303), Spb: *Schizosaccharomyces pombe* (NC_001326), Spr: *Saccharomyces pastorianus* (EU852811), Ss: *Saccharomyces servazzii* (AJ430679), St: *Strobilurus tenacellus* (X88000), Ta: *Trichosporon asahii* (AB175745), Tao: *Trichosporon asteroides* (AB175748), Td: *Trichosporon dulcitum* (AB175756), Ti: *Tilletia indica* (DQ993184), Tik: *Trichosporon inkin* (AB175764), Tj: *Trichosporon jirovecii* (AB175766), Tm: *Trichosporon moniliforme* (AB175773), To: *Trichosporon ovoides* (AB175777), Tp: *Trimorphomyces papilionaceus* (X85236), Um: *Ustilago maydis* (NC_008368), Uv: *Ustilaginoidea virense* (JN204426), Wm: *Williopsis mrakii* (X66594), Yl: *Yarrowia lipolytica* (NC_002659).

2 n.d, not determined means that position of this intron could not be defined because the *Cyt* b gene sequences with this intron was unavailable in database.

### Determination of Intron Positions and Phases

Information about intron location is important and the base for elucidating the dynamics of introns. In the present study, the relative location of an intron in the *Cyt b* gene is indicated by its “position” and “phase”. Intron position in this study refers to the position of codon at which the intron is inserted. Intron phase refers to the nucleotide position in a codon at which the intron is inserted. An intron is considered as phase “0” if it is inserted between two consecutive codons, “1” if inserted between the first and the second nucleotide of a codon, “2” if inserted between the second and the third nucleotide of a codon. The number, position and phase of introns in *Monilinia* spp. and *U. virens* were identified by comparing the gDNA and cDNA sequences. For *Cyt b* genes retrieved from the GOBASE database, the intron number, position and phase were determined as described below. To determine the locations of introns of the *Cyt b* genes, the deduced amino acid sequences of all *Cyt b* genes were aligned using the MegAlign program in the Software package DNASTAR (DNASTAR Inc., Nevada City, CA). For the locations of introns to be comparable in different *Cyt b* genes, their relative locations corresponding to the *Cyt b* gene in *U. virens* were considered as their locations. If the locations of two introns vary by fewer than 6 nucleotides, they are called “sliding introns”. The annotation for all sliding introns were checked manually as described in Figure S1. If possible, sliding introns were modified so that they could be adjusted to the same location. In most cases, the nucleotide at the 3′ end of a group I intron should be ‘G’ and the exonic base immediately upstream of a group I intron should be ‘T’ [42]–[44]. Introns with identical position are called common introns; they are referred to as unique intron if only a single intron is found at a certain position. Each intron is named after the abbreviation of species name followed by a number, with “1” as the most 5′ intron and the largest number as the most 3′ intron. For example, intron Mm3 refers to the 3^rd^ intron of the *Cyt b* gene from *M. mumecola*.

**Table 3:** Identification of homologues among fungal *Cyt b* gene introns^1^.

Homologue	Intron number	Intron position	Intron name										
1	9	69,67	Ad	Bf1	Mfc1	Mfg1	Ml1	Mm1	My1	Pa1	Uv1		
2	2	97	Mmj1	Mn1									
3	10	114,131	Hj1	Cb	Gz2	Mfc2	Mfg4	Ml2	Mm2	My2	Pm	Uv2	
4	2	126	Aj1	Pt2									
5	8	131	Bf2	Ef	Mmj2	Mn2	Nc1	Pa2	Pt3	Ptr1			
6	2	131	Cp1	Cm									
7	2	137	Ci	Cs									
8	2	139	Sc1	Sd2									
9	3	143	Ml3	Mm3	My3								
10	4	143	Bc	Co	Cp2	Mfn2							
11	6	164	Gz3	Ml4	Bf4	Mfg5	Nc2	Uv3					
12	2	164	My4	Mm4									
13	4	164	Gp1	Ta	Tad1	To							
14	11	168,169,208	Ac	Pn	Ab	Gz4	Mfc5	Mfg6	Ml5	Mm5	My5	Nf	Hj2
15	4	169	Gp2	Tad2	Ti	Tik							
16	2	169	Sc3	Sd4									
17	6	260	Gz5	Mfg7	Mfc6	My6	Mm6	Ml6					
18	3	275	Pa3	Pc	Uv6								
19	2	275	Ml7	My7									

1 the homology of *Cyt b* introns was identified by using the reciprocal BLAST to calculate the pairwise identity of introns with the cut off 1e-5. Two introns were considered as homologues if coverage >30% and pairwise identity >80%.

### Phylogenetic Analysis

Maximum parsimony (MP) method was used to construct phylogenetic trees. *Cyt b* gene cDNA sequences or intron sequences at position 164 were aligned with the program Clustal W in the software MEGA 4.0. The following settings were used to construct the phylogenetic trees: heuristic search using close neighbor interchange (CNI; level = 1) with initial trees generated by random addition (100 reps).

### Identification of Homology in *Cyt b* Introns

The homology of *Cyt b* introns was identified by using a reciprocal BLAST to calculate the pairwise nucleotide identity of introns. Algorithm BLASTN was used for the identity search in program BioPerl with default parameters and an E-value cutoff of 1e-05. To obtain an accurate estimate of global hit statistics, a tiling of high-scoring segment pairs (HSPs) onto either the subject or the query sequence was performed. Two introns were considered as homologues if coverage (the sum of HSP length/longer one in paired introns) >30% and average pairwise identity >80%.

**Figure 1:**
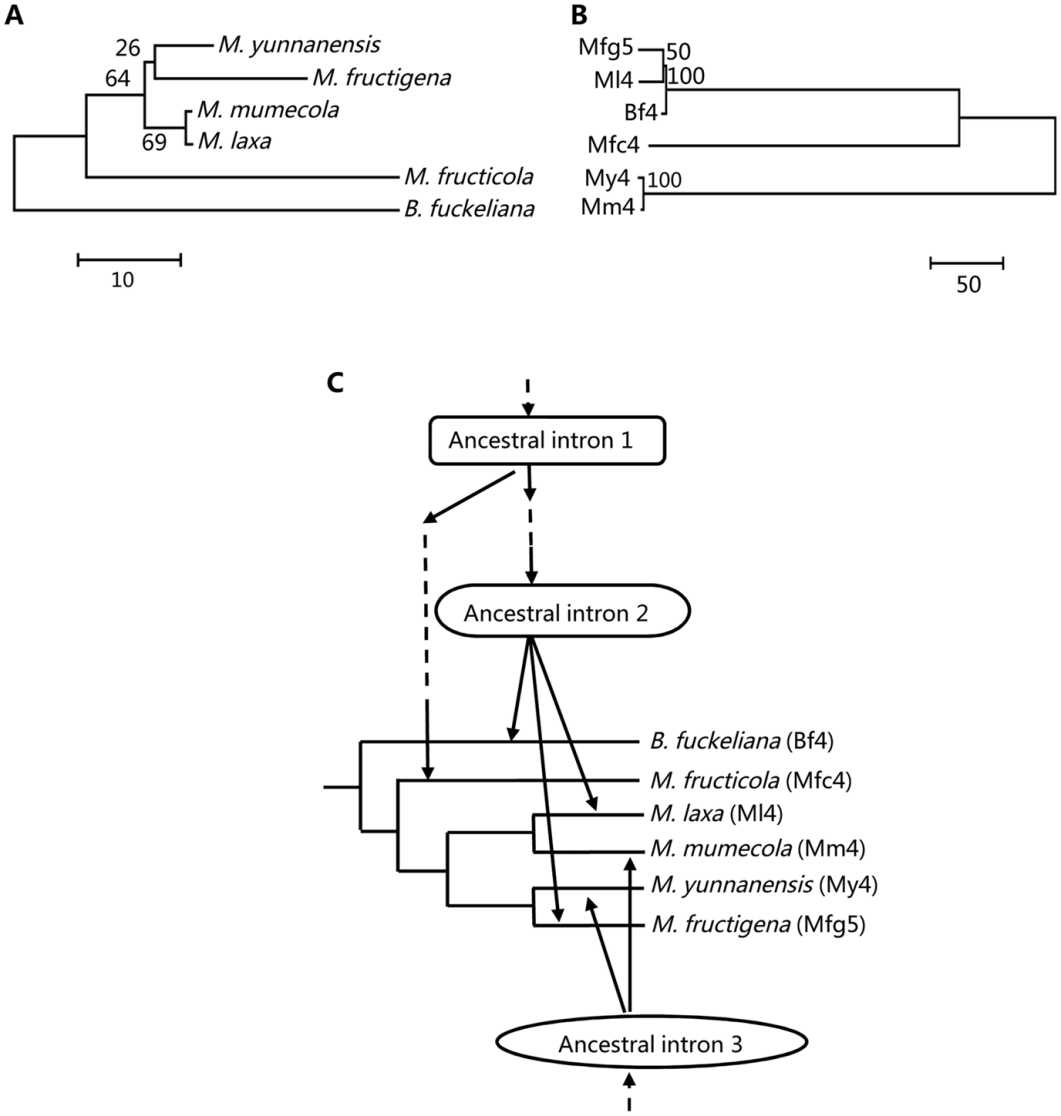
Maximum parsimony (MP) phylogenetic trees and schemata of intron gains in *Botryotinia* and *Monilinia* spp. (A) and (B), MP phylogenetic trees based on cDNA sequences and intron sequences at position 164 of the *Cyt b* gene from *Botryotinia* and *Monilinia* spp. The numbers at each node indicate the bootstrap (BS) values (N = 500) supporting individual branches. (C) Schemata of position 164 intron gains from lateral transfers in *Monilinia* species *M. fructicola*, *M. yunnanensis* and *M. mumecola*. Intron names are shown in parenthesis after the species name. The GenBank accession nos. of *Cyt b* genes in *Botryotinia* and *Monilinia* spp were AB428335 (*B. fuckeliana*), GQ304941 (*M. fructicola*), GU952817 (*M. laxa*), HM149254 (*M. fructigena*), HQ908793 (*M. yunnanensis*) and JN204425 (*M. mumecola*) which include the cDNA sequences and intron sequences at position 164.

**Figure 2:**
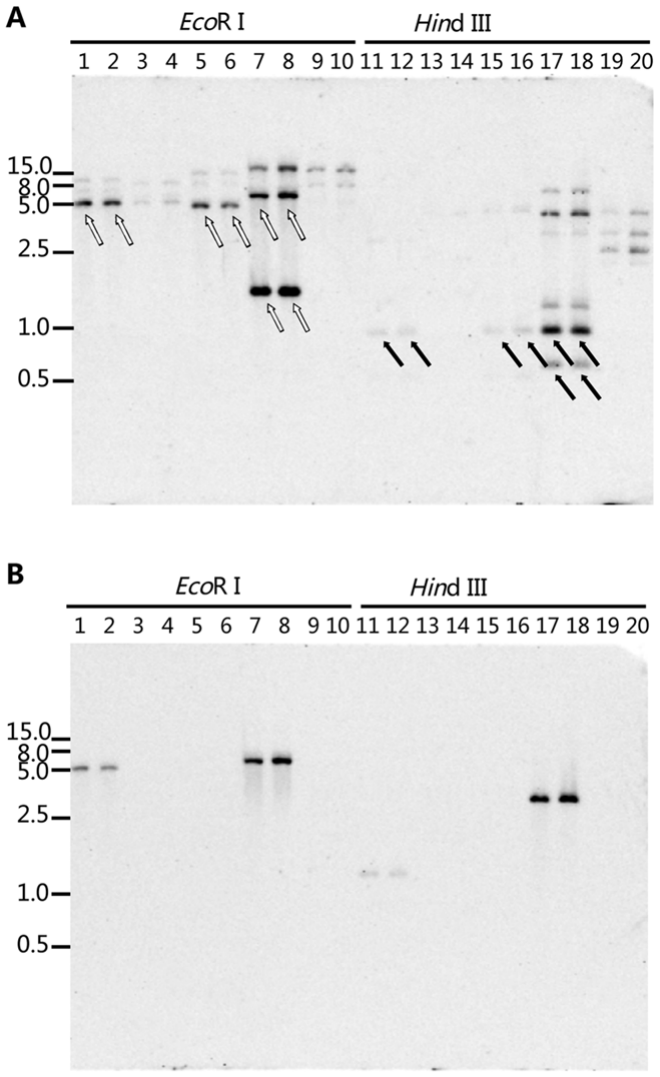
Identification of the *Cyt b* gene intron in *Monilinia* spp. Two restriction enzymes *Eco*R I and *Hin*d III were used to generate the restriction profiles. Digested genomic DNA was separated in 0.8% agarose gel, and the blot was hybridized with the My3 (A) and My4 (B) fragments. White and black arrows in (A) indicate the expected bands for *Eco*R I and *Hin*d III digestions. Lanes 1, 11 and 2, 12: *M. yunnanensis* isolates YKG10-64a and SM09-7c; 3, 13 and 4, 14: *M. fructigena* isolates SL10 and Mfg2-GE-A; 5, 15 and 6, 16*: M. laxa* isolates BEK-SZ and EBR ba11b; 7, 17 and 8, 18*: M. mumecola* isolates HWL10-11a and HWL10-20a; 9, 19 and 10, 20*: M. fructicola* isolates MPA14 and BM09-4a. The sizes (in kilobases) of marker DNA fragments are indicated on both sides (Wide Range DNA Marker on the left and DL 2000 DNA Marker on the right, TaKaRa Biotechnology (Dalin) Co., Ltd).

### Detection of *Cyt b* Introns in *Monilinia* and *Botryotinia* Genera by Southern Hybridization

Southern hybridization analysis was performed mainly as described previously [45]. In brief, DNAs were digested with *Eco*R I or *Hin*d III and separated in 1.0% agarose gels in 0.5×TBE buffer and transferred to Hybond N+ membranes (Amersham Pharmacia Biotech UK Limited, Buckinghamshire, UK). Intronic fragments were amplified by PCR reactions with the intron-specific primer pairs listed in Table S1. Thermal parameters for amplifying each fragment were as follows, 4 min at 94°C, 35 cycles of 40 sec at 94°C, 40 sec at 54°C, and 1.5 min at 72°C, a final elongation step of 72°C for 5 min was followed. All PCR products were gel-purified using the EasyPure Quick Gel Extraction Kit (TransGen Biotech, Beijing, China). Gel-purified PCR products were labeled with alkaline phosphate using AlkPhos Direct Labeling Reagents (Amersham Biosciences UK Limited). Hybridization was performed overnight at 55°C in Gene Images AlkPhos Direct hybridization buffer including 0.5 M NaCl and 4% blocking reagent (Amersham Biosciences UK Limited). Membranes were washed twice in primary buffer solution at 55°C for 10 min and then washed twice in secondary buffer solution at room temperature for 5 min. Target DNA was detected using the CDPstar reagent (Amersham International plc, Buckinghamshire, UK) and the signal from chemoluminescence was captured using ChemiDoc XRS+ Imager (Bio-Rad Laboratories Inc., California, USA).

## Results and Discussion

### Exon-intron Structure of *Cyt b* Gene in *Ustilaginoidea virens*

The entire *Cyt b* gene from *Ustilaginoidea virens* was 9216 bp in size and contained six introns. They were located at cDNA positions (5′–3′) 201, 393, 490, 506, 562 and 823 with sizes of 1594, 1180, 1223, 1775, 1325 and 949 bp respectively. The exon and intron sequences of isolate UV-8a are available in GenBank under accession number JN204426. In other organisms, relative locations of introns corresponding to the *Cyt b* gene in *U. virens* were considered as their locations.

### Skewed Distribution of *Cyt b* Introns in Different Kingdoms and Patchy Distribution of *Cyt b* Gene Introns in Fungi

*Cyt b* genes were obtained from 3,276 species of four eukaryotic kingdoms. Only 92 species, including six fungal pathogens (*Monilinia* spp. and *U. virens*), had introns (Table 1). In general, *Cyt b* gene introns were frequently present in organisms from the fungal kingdom and some lower plants such as green algae, liverworts, hornworts, true quillwort and moss, but generally absent or rare in the *Cyt b* genes from Chromista, Protozoa and Animalia. Therefore, the analysis of dynamics of *Cyt b* gene introns was focused on the fungal kingdom.

A total of 172 fungal introns from 69 *Cyt b* genes were identified and characterized extensively. Of the 172 introns investigated, the locations of 171 introns were determined as described in materials and methods, location of the intron En could not be defined because the *Cyt b* gene sequence with this intron was unavailable in the database. The introns of *Cyt b* genes varied dramatically in location and length, sometimes even between closely related species.

As a whole, fungal introns were found at 35 positions in *Cyt b* genes, showing a wide and patchy distribution. Of the 35 positions, 19 had common introns (present in at least two genes) and 16 had unique introns (present in only one gene). The intron numbers at individual positions varied from a single representative at 16 different positions to 32 different introns at position 131 (Table 2). The introns at the 16 common positions contained 2 (positions 93, 137 etc.) to 32 (position 131) introns in distinct organisms. The observation that different introns located at same positions are consistent with the fact that mobile introns tend to insert into specific locations because they are limited by the nature of the cutting/recognition sequences of their encoded homing endonucleases [46]–[47].

### Frequent Intron Losses in Fungal *Cyt b* Genes

To understand the relationship between *Cyt b* introns, the homology of 171 fungal *Cyt b* gene introns with known positions was analyzed. Theoretically, hit scores below e-value threshold (1e-05) have a certain chance of being homologous. However, the sum of HSPs of many hits only covered a limited part of either introns. Such introns should not be real homologues but just contained certain conserved regions. Thus, in the present study, only introns meeting additional criteria (coverage >30% and average pairwise identity >80%) were considered homologues.

Altogether 19 homologues were identified (Table 3). As expected, most homologous introns were located at the same locations. Many homologous introns were present at the same position in relatively distinct species but absent in closely related species, indicating that frequent intron loss in *Cyt b* genes had occurred. For example, at position 169, homologous introns were frequently detected from phylum Ascomycota (at least from 3 classes, Sordariomycetes (Gz4), Eurotiomycetes (Ab, Nf) and Leotiomycetes (Mfc5, Mfg6, Mm5, Ml5 and My5), indicating that these introns were vertically inherited from a common ancestral intron before the divergence of these classes. These highly homologous introns were detected in all of the investigated species of *Monilinia* genus but not from the species *B. fuckeliana* of the same Sclerotiniaceae family, indicating that the intron at position 169 was recently lost in *B. fuckeliana* (after the divergence of *Monilinia* and *Botryotinia* genera). Intron loss events were also observed at position 67: homologous introns were detected in species from the Leotiomycetes (Bf1, Mfc1, Mfg1, Mm1, Ml1 and My1) and Sordariomycetes (Uv1, Pa1), but not in the *G. zeae* species which is in the same order Hypocreales with *U. virens* under Sordariomycetes class. Besides positions 67 and 169, similar intron loss events were observed at least at positions 131, 143, 164, 260 (Table 3). Such frequent intron loss events may help explain why more than 97% of investigated species contained intron-free *Cyt b* genes and more than half of the *Cyt b* genes containing introns had only one or two of them (Table 1).

### Frequent Intron Gains in Fungal *Cyt b* Genes

Analysis of homology of fungal *Cyt b* introns indicated that some introns located at the same common position did not show similarity though the corresponding species are closely related. For instance, at position 164, introns Ml4 and Mfg5 were not homologous with introns My4 and Mm4 (Table 3), although they are from species of the same genus. These unrelated introns might have recently inserted at the same gene position of different organisms independently. The highly divergent sequences between introns at the same position were observed in closely related species, strongly indicating the high mobility of these introns.

Some introns at the same common position were more similar in distantly related species than in closely related species. As shown in Table 3 and Figure 1A, at position 164 intron Mm4 of *M. mumecola* did not show similarity with intron Ml4 in *M. laxa* although the two species are closely related (Figure 1A). Similarly, intron My4 in *M. yunnanensis* was not homologous with intron Mfg5 in *M. fructigena* although their corresponding *Cyt b* coding sequence had 99.1% nucleotide identity. On the other hand, introns Mm4 and My4 in two distantly related *Monilinia* spp were highly homologous and had 99.4% nucleotide identity (Figure 1B). Such highly similar intron sequences in distantly related species suggests that the introns were gained independently, likely through lateral transfers after speciation of the *Monilinia* genus (Figure 1C), though how the transfers occurred is still unclear. Intron Mfc4 was also not homologous with other introns, suggesting that it was gained in a similar fashion (Table 3, Figure 1C). By contrast, introns Ml4 and Mfg5 were homologous with other introns Bf4, Gz3, Nc2 and Uv3, indicating that those introns were inherited vertically from a common ancestral intron.

Most of the mitochondrial group I introns could encode LAGLIDADG or GIY-YIG DNA endonucleases, which play a role in the transfer and site-specific integration (“homing”) of the intron (Lazowska, Jacq et al. 1980; Lambowitz and Perlman 1990; Pellenz, Harington et al. 2002). In the present study, most introns at common positions contained the LAGLIDADG endonuclease coding sequence (e.g. Mm4, Ml4, Mfc4 etc.) or the GIY-YIG endonuclease coding sequence (e.g. Bf2, Pa2, My2) and thus should be able to move by lateral transfer. Homing endonuclease genes (HEGs) are disproportionately common in mitochondria and chloroplasts of eukaryotes. The first HEG discovered was ω (I-SceI) in the large subunit rRNA gene in the mitochondria of the yeast *Saccharomyces cerevisiae* [48]–[49]. The study of the evolutionary dynamics of HEGs showed that they are by regularly moving to new species through horizontal transmission, which starts the process of invasion, spread and fixation all over again, and the long-term persistence of HEGs in a single population relies on low transmission rates and a positive correlation between transmission efficiency and toxicity [16], [46], [50]. The introns in *Cyt b* genes of *Monilinia* spp. contained HEGs which should be beneficial for introns to specific recognition sites by horizontal transmission. Therefore, the high number of *Cyt b* introns in *Monilinia* spp. appeared to be a result of recent intron gain events and this directly caused *Monilinia Cyt b* genes to be the largest ones in eukaryotes.

### Intron Loss-and-gain Events could be Mediated by Transpositions

As shown in Table 3, some introns within individual homologues are from different positions. For instance in homologue 1, intron Ad was homologous with introns Bf1, Mfc1 etc, but it was located at position 69, while other homologous introns were located at position 67, suggesting that Ad was transposed to a new position during evolution. Introns Pn, Ac (position 168) and Hj2 (position 208) in homologue 16 were also transposed to the new positions from position 169 where their six homologous introns (e.g. Mfc5, Gz4 etc) were located. Similarly, intron Hj1 has been transposed to position 114 from position 131 where its nine homologues were located. Pronounced transposition event was observed in species *Volvox carteri* (homologue 9), where the homologous introns Vc1 and Vc2 were located at different positions in the same gene. These observations suggested that the frequent gain-and-loss of introns occurred during evolution of *Cyt b* genes and some of the *Cyt b* introns might be TEs because they can move to new sites and undergo deletion or amplification in genomes.

Hybridization experiments were performed to investigate transposability of *Cyt b* intron sequences in the genome of *Monilinia* species. Intron My3 from *M. yunnanensis* is located at position 143, southern hybridization using My3 as probe showed expected bands (those with arrows in lanes 1, 2 and 11, 12 in Figure 2A) and additional bands (those without arrows) in *M. yunnanensis* isolates. Expected bands were detected in *M. laxa* (with arrows in lanes 5, 6 and 15, 16) and *M. mumecola* (lanes 7, 8 and 17, 18 in Figure 2A) as My3 was homologous with introns Ml3 and Mm3, while unexpected bands (without arrows) were also detected. Bands were even detected from *M. fructigena* (without intron at position 143) and *M. fructicola*, both species did not have homologous introns of My3 in their *Cyt b* genes. These results indicate that multiple copies of introns My3, Ml3 and Mm3 exist in the genomes of *M. yunnanensis*, *M. laxa* and *M. mumecola*, as well as in *M. fructigena* and *M. fructicola*. These introns were repetitive elements, a characteristic for TEs. Mfg2 was also confirmed to be a repetitive intron (Figure S2A). The multiple copies of introns could either be mitochondrial or nuclear since we used the total genomic DNAs in the experiments. By contrast, My4 and Ml4 were single copy elements (Figure 2B, Figure S2B), thus should not be considered TEs.

### Intron Gains can have a Profound Impact on the Phenotype

Single amino acid changes in the *Cyt b* gene can confer QoI fungicide resistance in plant pathogenic fungi. For instance, in the plant pathogens *B. graminis*, *Sphaerotheca fuliginea*, *Mycosphaerella fijiensis* and *Venturia inaequalis*, fungicide resistance is conferred by a single point mutation in the *Cyt b* gene, leading to an amino acid substitution at position 143 from glycine to alanine (G143A) (Gisi, Sierotzki et al. 2002; Sierotzki, Schlenzig et al. 2002). The presence of an intron at the position 143 has been shown to prevent the formation of the G143A mutation in some pathogens, because mutations in this codon would interfere with intron splicing and consequently prevent the formation of an active *Cyt b* gene [31], [51]–[52]. A similar mechanism was documented in *S. cerevisiae*, where an exon mutation at the second nucleotide upstream of the splice site of intron 4 in the *Cyt b* gene (COB4) did not permit correct splicing of the pre mRNA [53]–[54].

Two different *Cyt b* alleles were discovered, one with and one without an intron at position 143 in *B. fuckeliana*. Southern hybridization using intron Bf3 as probe showed that this intron was absent from the entire genome of isolates lacking the intron at position 143 (Figure S2C). Homology analysis of the Bf3 showed that it was not homologous with any of introns including those at the same position in closely related *Monilinia* spp. (Table 3). Therefore, the Bf3 should be laterally gained at position 143 in isolates with Bf3 rather than lost in the isolates without the intron. Since only *B. fuckeliana* isolates lacking the intron Bf3 have the G143A mutation and are highly resistant to QoI fungicides (Banno, Yamashita et al. 2009; Ishii, Fountaine et al. 2009), the Bf3 gained event directly influenced the development of the QoI fungicide resistance. Once the isolates gained the Bf3, they lost the potential ability to develop the fungicide resistance based on the G143A mutation. Pathogens did not get any visible benefit from such an intron gain event but had negative influence for the development of fungicide resistance, suggesting that it was just one of many intron gain-and-loss events occurring neutrally and the intron gain-and-loss events were not necessarily beneficial to their host organisms.

## Supporting Information

Table S1 Primers used for intron-specific PCR amplification
(DOC)

Figure S1 Modification of the sliding intron position or phase. (A) The phase of intron Sc5 was modified based on one nucleotide shifting of the exon/intron sequence. (B) The phase of intron Gi3 was modified based on two nucleotide shifts that altered one codon. (C) Sliding introns can’t be modified to same position or phase. Lowercase letters indicate intron sequences, and uppercase letters represent the flanking exon sequences, the larger uppercase letters represent the codon at which individual introns located. If introns have different positions or phases, they are considered as different locations. The locations of introns were modified slightly from original information for some sliding introns. For example, the intron Sc5 at position 270 was a phase 0 intron based on the database information, but one nucleotide shifting of exon/intron sequence could make it have same phase with other introns at this position (such as Cbs2(2), Cc(2) etc. in table 2), as well as have common group I intron features (A). The intron Gi3 at position 164 was a phase 0 intron, but two nucleotide shifts of exon/intron sequence would fulfill the above mentioned criteria, though one exonic codon was altered (B). In contrast, the same criteria did not allow sliding the introns (C).
(TIF)

Figure S2 Identification of the *Cyt b* gene introns in *Monilinia* spp and *B. fuckeliana*. Two restriction enzymes *Eco*R I and *Hin*d III were used to generate the restriction profiles. Digested genomic DNA was separated in 0.8% agarose gel, and the blot was hybridized with the Mfg2 (A), Ml4 (B) and Bf3 (C) fragments. Lanes 1, 11 and 2, 12: *M. yunnanensis* isolates YKG10-64a and SM09-7c; 3, 13 and 4, 14: *M. fructigena* isolates SL10 and Mfg2-GE-A; 5, 15 and 6, 16*: M. laxa* isolates BEK-SZ and EBR ba11b; 7, 17 and 8, 18*: M. mumecola* isolates HWL10-11a and HWL10-20a; 9, 19 and 10, 20*: M. fructicola* isolates MPA14 and BM09-4a in *A* and *B*. For *B. fuckeliana* in C, 1, 5, isolate RhosimBC-4; 2,6, isolate PeachBC-1; 3,7, isolate GarlicBC-78; 4,8, isolate ViotriBC-1. The sizes (in kilobases) of marker DNA fragments are indicated on both sides (Wide Range DNA Marker on the left and DL 2000 DNA Marker on the right, TaKaRa Biotechnology (Dalin) Co., Ltd).
(TIF)
