# Supplementary material for: Frequent Gain and Loss of Introns in Fungal Cytochrome b Genes
Source: PLoS One. 2012 Nov 7;7(11):e49096. doi: 10.1371/journal.pone.0049096 (PMC3492308; doi:10.1371/journal.pone.0049096)
Supplement: Table S1 — Primers used for intron-specific PCR amplification (DOC) [file pone.0049096.s001.doc]

Table S1. Primers used for intron-specific PCR amplification

| Primer Name | Primer Sequence (5′ → 3′) | Description |
| --- | --- | --- |
| Bc_Intron3-F  Bc_Intron3-R | acgaataggaccgcatcatc  Cttcaggcatgagaccctgt | To amplify the intron Bf3 |
| MG_Intron2F  MG_Intron2R | Atttggtattgggcaggttg  taaccgccaaggtattacgc | To amplify the intron Mfg2 |
| MY_Intron3F  MY_Intron3R | Tgtggggaacagataggatc  accctgtgcagtacacttatc | To amplify the intron My3 |
| MC_Intron3F  MC_Intron3R | Ttatttcgtgcctcacatgc  caccgatggagcagtctgta | To amplify the intron Mfc3 |
| ML_Intron4F  ML_Intron4R | ggtatggtggatggagatgg cttggttgtagggcattcgt | To amplify the intron Ml4 |
| MY_Intron4F  MY_Intron4R | tcggatccgtctaccatagc  ggtgggaaaagcattcatgt | To amplify the intron My4 |
