# Supplementary figures and images for: Frequent Gain and Loss of Introns in Fungal Cytochrome b Genes
Source: PLoS One. 2012 Nov 7;7(11):e49096. doi: 10.1371/journal.pone.0049096 (PMC3492308; doi:10.1371/journal.pone.0049096)

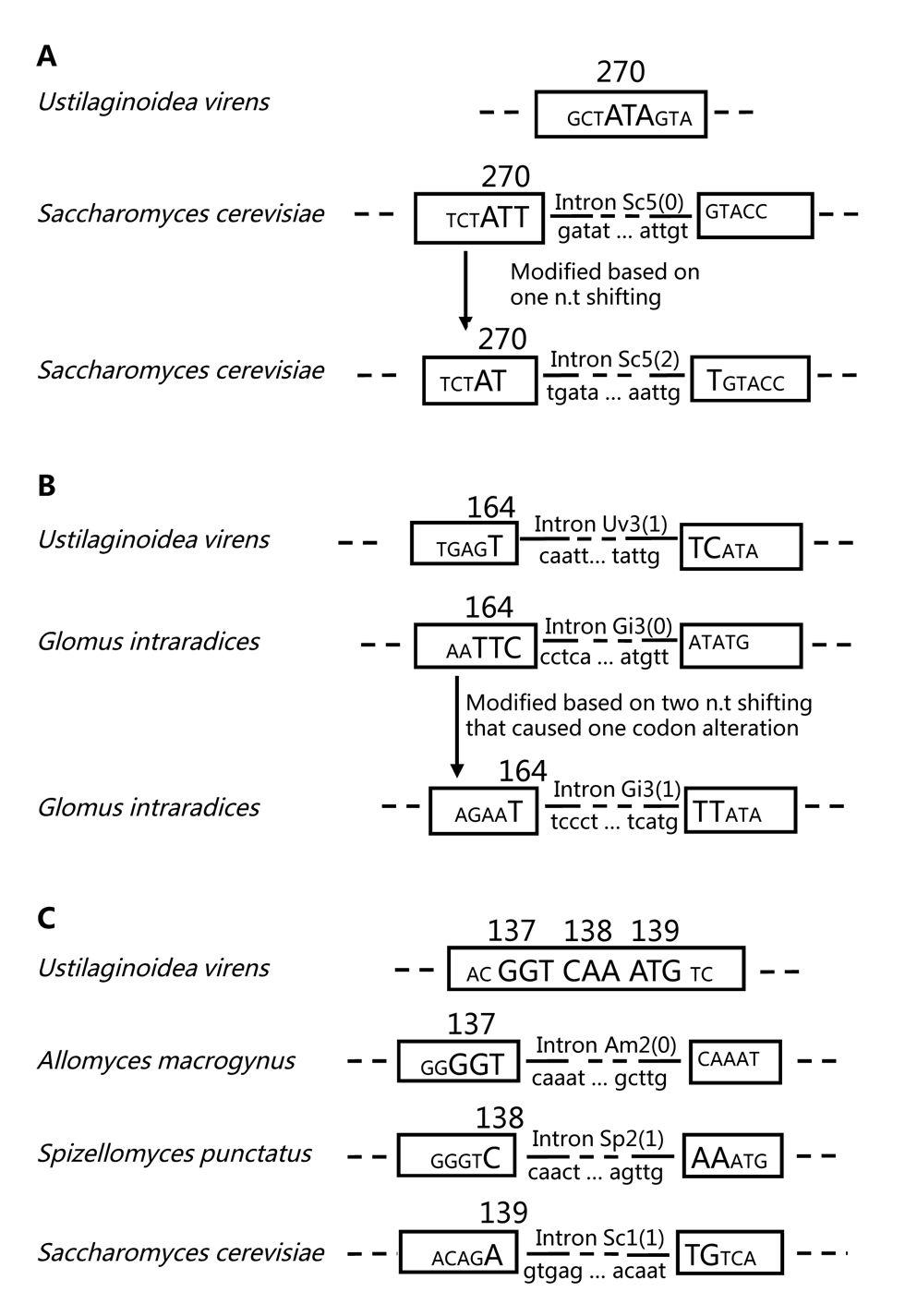

Supplement: Figure S1 — Modification of the sliding intron position or phase. (A) The phase of intron Sc5 was modified based on one nucleotide shifting of the exon/intron sequence. (B) The phase of intron Gi3 was modified based on two nucleotide shifts that altered one codon. (C) Sliding introns can’t be modified to same position or phase. Lowercase letters indicate intron sequences, and uppercase letters represent the flanking exon sequences, the larger uppercase letters represent the codon at which individual introns located. If introns have different positions or phases, they are considered as different locations. The locations of introns were modified slightly from original information for some sliding introns. For example, the intron Sc5 at position 270 was a phase 0 intron based on the database information, but one nucleotide shifting of exon/intron sequence could make it have same phase with other introns at this position (such as Cbs2(2), Cc(2) etc. in table 2), as well as have common group I intron features (A). The intron Gi3 at position 164 was a phase 0 intron, but two nucleotide shifts of exon/intron sequence would fulfill the above mentioned criteria, though one exonic codon was altered (B). In contrast, the same criteria did not allow sliding the introns (C). (TIF) [file pone.0049096.s002.tif]

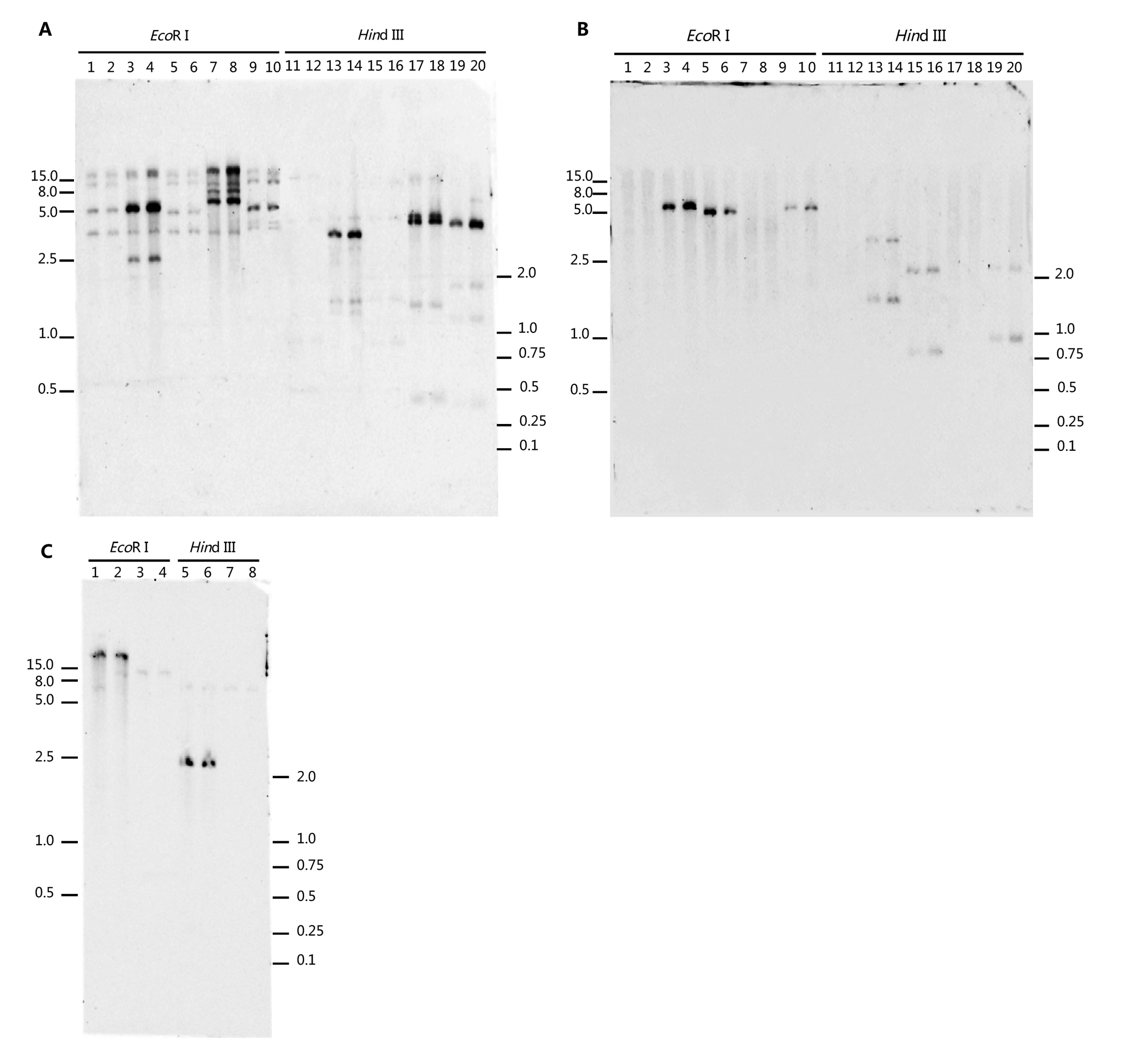

Supplement: Figure S2 — Identification of the Cyt b gene introns in Monilinia spp and B. fuckeliana. Two restriction enzymes EcoR I and Hind III were used to generate the restriction profiles. Digested genomic DNA was separated in 0.8% agarose gel, and the blot was hybridized with the Mfg2 (A), Ml4 (B) and Bf3 (C) fragments. Lanes 1, 11 and 2, 12: M. yunnanensis isolates YKG10-64a and SM09-7c; 3, 13 and 4, 14: M. fructigena isolates SL10 and Mfg2-GE-A; 5, 15 and 6, 16: M. laxa isolates BEK-SZ and EBR ba11b; 7, 17 and 8, 18: M. mumecola isolates HWL10-11a and HWL10-20a; 9, 19 and 10, 20: M. fructicola isolates MPA14 and BM09-4a in A and B. For B. fuckeliana in C, 1, 5, isolate RhosimBC-4; 2,6, isolate PeachBC-1; 3,7, isolate GarlicBC-78; 4,8, isolate ViotriBC-1. The sizes (in kilobases) of marker DNA fragments are indicated on both sides (Wide Range DNA Marker on the left and DL 2000 DNA Marker on the right, TaKaRa Biotechnology (Dalin) Co., Ltd). (TIF) [file pone.0049096.s003.tif]
